# The Processes and Mechanisms of Cardiac and Pulmonary Fibrosis

**DOI:** 10.3389/fphys.2017.00777

**Published:** 2017-10-12

**Authors:** Lucy A. Murtha, Michael J. Schuliga, Nishani S. Mabotuwana, Sean A. Hardy, David W. Waters, Janette K. Burgess, Darryl A. Knight, Andrew J. Boyle

**Affiliations:** ^1^School of Medicine and Public Health, University of Newcastle, Callaghan, NSW, Australia; ^2^Hunter Medical Research Institute, New Lambton Heights, NSW, Australia; ^3^School of Biomedical Sciences and Pharmacy, University of Newcastle, Callaghan, NSW, Australia; ^4^Department of Pathology and Medical Biology, Groningen Research Institute for Asthma and COPD, W. J. Kolff Research Institute, University of Groningen, University Medical Center Groningen, Groningen, Netherlands; ^5^Respiratory Cellular and Molecular Biology Group, Woolcock Institute of Medical Research, Glebe, NSW, Australia; ^6^Discipline of Pharmacology, University of Sydney, Sydney, NSW, Australia; ^7^Department of Anesthesiology, Pharmacology, and Therapeutics, University of British Columbia, Vancouver, BS, Canada; ^8^Department of Medicine, University of Western Australia, Perth, WA, Australia; ^9^Research and Innovation Conjoint, Hunter New England Health, Newcastle, NSW, Australia

**Keywords:** cardiac fibrosis, pulmonary fibrosis, heart failure, myocardial infarction, idiopathic pulmonary hypertension, acute respiratory distress syndrome, heart, lung

## Abstract

Fibrosis is the formation of fibrous connective tissue in response to injury. It is characterized by the accumulation of extracellular matrix components, particularly collagen, at the site of injury. Fibrosis is an adaptive response that is a vital component of wound healing and tissue repair. However, its continued activation is highly detrimental and a common final pathway of numerous disease states including cardiovascular and respiratory disease. Worldwide, fibrotic diseases cause over 800,000 deaths per year, accounting for ~45% of total deaths. With an aging population, the incidence of fibrotic disease and subsequently the number of fibrosis-related deaths will rise further. Although, fibrosis is a well-recognized cause of morbidity and mortality in a range of disease states, there are currently no viable therapies to reverse the effects of chronic fibrosis. Numerous predisposing factors contribute to the development of fibrosis. Biological aging in particular, interferes with repair of damaged tissue, accelerating the transition to pathological remodeling, rather than a process of resolution and regeneration. When fibrosis progresses in an uncontrolled manner, it results in the irreversible stiffening of the affected tissue, which can lead to organ malfunction and death. Further investigation into the mechanisms of fibrosis is necessary to elucidate novel, much needed, therapeutic targets. Fibrosis of the heart and lung make up a significant proportion of fibrosis-related deaths. It has long been established that the heart and lung are functionally and geographically linked when it comes to health and disease, and thus exploring the processes and mechanisms that contribute to fibrosis of each organ, the focus of this review, may help to highlight potential avenues of therapeutic investigation.

## Introduction

The underlying molecular and cellular events of fibrotic diseases share many functional similarities, despite differences in etiology and clinical outcome. At its core, chronic fibrosis is pathological and continuous wound-healing which can effect multiple organ systems including but not limited to the heart, lung, kidney, liver, and skin. Although fibrosis is an initially beneficial process of repair, continuous accumulation of fibrotic proteins leads to permanent tissue remodeling and significant organ impairment. The maladaptive response to injury or challenge results in an excessive accumulation of extracellular matrix (ECM) proteins, and is mediated by fibroblasts and myofibroblasts. Elements of the innate and adaptive immune systems also have important roles, facilitating fibroblast transdifferentiation and ridding the damaged tissue of apoptotic cells and ECM debris (Zymek et al., 2006; Wynn and Ramalingam, 2012; Frangogiannis, 2014). Fibrosis typically presents as interstitial (reactive) or replacement (reparative) fibrosis. Interstitial fibrosis refers to a chronic and progressive expansion or scarring of the interstitium as a means of maintaining organ function. Replacement fibrosis is activated by an injury, and is a reparative process in which dead cells are replaced with a collagen based scar (Krenning et al., 2010; Biernacka and Frangogiannis, 2011; Shinde and Frangogiannis, 2014). Human organ fibrosis has been estimated to contribute to 45% of all-cause human death, and yet effective treatments are currently lacking. The molecular events and processes which contribute to the onset and development of fibrosis must be elucidated in order to develop novel efficacious treatments. Given the intimate connection between the heart and lung, this review will focus on the pathophysiology of fibrosis in these two organs.

## Fibrotic disease of the heart

Cardiovascular disease (CVD) is the single leading cause of death worldwide. The World Health Organization estimates that 31% of all deaths each year are attributed to CVD (17.5 million per annum; World Health Organization, 2014). Although, the rate of CVD-related deaths has dramatically declined over the past four decades (>28%), the burden of disease is still high (Mozaffarian et al., 2016). The reduced incidence of CVD-related deaths is largely due to significant risk factor reduction and improved post-event management. Cardiac fibrosis is implicated in almost all forms of CVD. Initially cardiac fibrosis is thought to be an adaptive, protective mechanism, however over time fibrosis leads to irreversible ventricular remodeling and significantly impaired heart function. Two common forms of CVD in which fibrosis plays a major role are myocardial infarction and heart failure.

### Myocardial infarction

Greater than a third of CVD related deaths are attributed to myocardial infarction. Myocardial infarction is the result of a blocked coronary artery, and is most common in those aged over 65 years. Without the capacity for cellular regeneration, the cardiac tissue must remodel at the cellular level in order to meet the physiological demands of the system. The signaling pathways which are activated following myocyte death initiate acute reparative changes in the heart which can be broken into initial and late phases. The initial phase involves the replacement of necrotic tissue with fibrotic scar formation, elongation of myocytes and thinning of the infarct zone. The volume of the ventricle increases as an adaptive mechanism to maintain normal cardiac output and stroke volume (Sutton and Sharpe, 2000; Konstam et al., 2011). The late phase of ventricular remodeling involves hypertrophic myocyte elongation in the non-infarcted areas, increased wall mass, and chamber enlargement. The ventricle morphologically elongates and the performance begins to decline. The late phase will progress indefinitely and ultimately results in heart failure (Konstam et al., 2011). Replacement of damaged muscle mass with fibrotic tissue is entirely dependent on the physiological functioning of cardiac fibroblasts (Furtado et al., 2016).

### Heart failure

Improved medical care has resulted in a larger population surviving CVD events, and therefore, the prevalence of secondary complications such as heart failure has increased. Currently, one in five people globally will develop heart failure and survival estimates at 5 and 10 years post-diagnosis are 50 and 10%, respectively (Taylor et al., 2012). Heart failure is defined by the American Heart Association as “a complex clinical syndrome that results from any structural or functional impairment of ventricular filling or ejection of blood” (Yancy et al., 2013). Heart failure can be broadly classified as systolic heart failure or diastolic heart failure. Systolic heart failure is the result of a reduced ejection fraction and is characterized by reduced efficiency in the contraction and ejection of blood by the myocardium. Increased collagen deposition in systolic dysfunction is largely due to the need for cardiomyocyte replacement after cardiac damage. This increased ventricular stiffness results in reduced contraction and cardiac output, and a reduced systemic perfusion capacity (Khan and Sheppard, 2006). Disruption of myocardial excitation-contraction coupling, uncoordinated contraction of cardiomyocyte bundles, disrupted endomysial homeostasis, and sliding displacement of cardiomyocytes resulting in ventricular dilation have been proposed as mechanisms of fibrosis-induced systolic dysfunction (Biernacka and Frangogiannis, 2011). In diastolic heart failure, the ejection fraction is preserved, however there is a disturbance in the accommodation of blood volume during diastole at low filling pressures. Diastolic heart failure is largely a consequence of impaired ventricular relaxation or increased myocardial stiffness caused by excessive cardiac fibrosis (Gomes et al., 2013). The mechanisms underlying diastolic heart failure involve abnormal ECM composition and cell-ECM interactions, as well as alterations in the myocyte cytoskeleton, both of which impair the passive properties of the ventricular wall (Gomes et al., 2013).

## Fibrotic disease of the lungs

Fibrosis is also a characteristic of many respiratory diseases that differ markedly in etiology, phenotype, and symptoms. Fibrosis can occur in the airway wall, as in the larger airways in asthma (Boorsma et al., 2014) and around the small airways in chronic obstructive pulmonary disease (Jones et al., 2016), or the lung parenchyma, as in idiopathic pulmonary fibrosis (IPF) and acute respiratory distress syndrome (ARDS). The fibrosis in IPF is particularly prominent, causing irreversible lung scarring and respiratory failure (Boorsma et al., 2014; Burnham et al., 2014; Wuyts et al., 2014).

### Idiopathic pulmonary fibrosis

Interstitial lung diseases including IPF are characterized by abnormalities between the capillaries and alveolar spaces of parenchymal tissue. In IPF, the aberration is a relentlessly progressive fibrosis that causes irreversible damage to the lung structure and function (Ryu et al., 2014). Patients with IPF have a median survival of only 2–5 years from diagnosis. The prevalence of IPF in the United States is ~40 cases per 100,000, and accounts for over 16,000 deaths per year (Lynch et al., 2016; Raimundo et al., 2016). The health burden of IPF in other developed and developing countries is no less significant. IPF is diagnosed primarily in the elderly, and more so in males and/or patients with a history of smoking (Smith et al., 2013; Wuyts et al., 2014). Histologically, the spatially heterogeneous pattern of fibrosis in IPF, referred to as “usual interstitial pneumonia” (UIP), comprises architectural distortion, interstitial thickening, presence of fibroblastic foci and honeycombing cystic remodeling (Rabeyrin et al., 2015). UIP is primarily associated with IPF, but also occurs in other less common interstitial lung diseases, including connective tissue-disease associated interstitial lung diseases and chronic hypersensitivity pneumonitis (Wuyts et al., 2014). Whilst of unknown etiology (hence the term “idiopathic”), IPF is now widely considered to be initiated by persistent injuries to the alveolar epithelium (Selman et al., 2004). Furthermore, for reasons that remain incompletely understood, the subsequent injury-repair response to alveolar injury is highly dysregulated, resulting in the persistence of fibroblasts and the excessive accumulation of ECM proteins that scar the lung in IPF. Following an acute exacerbation, IPF patients display additional damage known as diffuse alveolar damage, which is characterized by patterns of inter and intra-alveolar accumulation of fibroblasts and ECM. This additional damage involves regions of lung previously spared from fibrosis and scarring (Papiris et al., 2010; Bhatti et al., 2013). Coupled with the existing UIP pattern, these exacerbations, typically of unknown cause, accelerate the normally gradual decline of respiratory function into a rapidly deteriorating state.

### Acute respiratory distress syndrome

ARDS is caused by injuries to the alveolar capillary barrier, comprising an acute inflammation phase and a subsequent fibroproliferation phase leading to interstitial fibrosis (Frenzel et al., 2011). ARDS afflicts ~200,000 people annually in the US, accounting for about 75,000 deaths per annum (Rubenfeld et al., 2005). ARDS prevalence and mortality increases with age, and is higher in men than women (Rubenfeld et al., 2005; Cheifetz, 2016; Cochi et al., 2016). ARDS is initiated by increases in systemic inflammation of pulmonary or extra-pulmonary origin. Insults range from direct forms, including viral, fungal, or bacterial pulmonary infections to indirect forms such as sepsis, peritonitis, or severe acute pancreatitis (Meduri and Eltorky, 2015). Injury is followed by an acute inflammation phase with leak of edema fluid into the interstitium and alveoli spaces. Resolution occurs if the damage from the initial injury is not severe and ongoing, and when repair processes which restore alveolar capillary barrier integrity occur in a timely manner. Any adaptive repair response involves a degree of fibroblast activation and migration to guide tissue repair by supporting epithelial and vascular regeneration. However, in many cases of ARDS, an excessive and persistent fibro-proliferation response occurs leading to interstitial fibrosis (Frenzel et al., 2011). Mechanical injury as a consequence of low tidal volume ventilation treatment strategies heightens the maladaptive fibrotic repair response. ARDS mortality, which is ~25% in the US, is strongly associated with the magnitude of pulmonary fibrosis (Rocco et al., 2009). Histologically, the pattern of tissue damage in ARDS is referred to as diffuse alveolar damage, which is differentiated in exudative and fibro-proliferative phases (Meduri and Eltorky, 2015). The exudative phase shows spatially uniform alveolar damage, fibrin deposition (hyaline membranes) along the alveolar wall, lung edema and hemorrhage. The later phase exhibits patterns of inter and intra-alveolar accumulation of fibroblasts and ECM.

## Molecular and cellular fibrotic mechanisms

### Fibroblasts

Fibroblasts are a spindle shaped population of mesenchymal cells that are found in the connective tissue of most organs (Shinde and Frangogiannis, 2014). Physiological injury or insult activates fibroblasts near the site of injury to efficiently undergo dynamic phenotypic changes that aide in scar formation (Shinde and Frangogiannis, 2014). Recent data has demonstrated that there is extensive heterogeneity in fibroblasts from different tissues, as well as those from the same tissue type (Lajiness and Conway, 2014). Fibroblasts are regarded primarily as the producers of the ECM, and do so via their extensive endoplasmic reticulum. The ECM scaffold they produce serves multiple roles including the support of adjacent cells, cell signaling, and cell adhesion (Hynes, 2009; Frantz et al., 2010; Lajiness and Conway, 2014; Shinde and Frangogiannis, 2014). Maintaining tissue homeostasis under normal conditions involves a fine balance in the expression of the ECM network constituents (Meneghin and Hogaboam, 2007). Under pathological conditions, fibroblast activation and proliferation is augmented, leading to increased production of ECM at the site of injury. Over-production of ECM components is a commonly observed hallmark of fibrotic diseases, and ultimately results in a severely scarred, inflexible and functionally compromised organ.

The origin of fibroblasts in both the heart and lung remain unclear. In the heart it has been proposed that they may originate either from local interstitial fibroblasts or from circulating precursor cells from the bone marrow (Yano et al., 2005). In the lung, the origins of the fibroblasts may also be derived from resident interstitial fibroblasts or circulating bone marrow-derived fibrocytes, or could be derived from epithelial-mesenchymal transdifferentiation (Mubarak et al., 2012; Maharaj et al., 2013; Burnham et al., 2014). Aside from epithelial cells, transdifferentiation of pleural mesothelial cells also possibly contribute to the increased number of myofibroblasts, particularly as fibrosis in IPF emanates from the sub-pleura region (Mubarak et al., 2012). Regardless of source, fibroblasts contribute to fibrosis by proliferating and differentiating into collagen producing, contractile myofibroblasts as observed in cardiac and pulmonary pathologies.

### Fibrotic process in the heart and lung disease

Myocardial infarction induced fibrosis occurs in three distinct and controlled phases: the inflammatory, proliferative, and maturation phases (Zymek et al., 2006; Frangogiannis, 2014; Table 1). Myocardial necrosis induced by sudden acute death of cardiomyocytes initiates the inflammatory phase, in which innate immune pathways are activated, inflammatory mediators are released, leukocytes are recruited to the infarcted site and neutrophils are activated (Zymek et al., 2006; Frangogiannis, 2014). The primary inflammatory phase is then actively suppressed to prepare the site for the proliferative phase. In this phase, mononuclear cells and macrophage subpopulations secrete growth factors that recruit and activate reparative cells including fibroblasts and myofibroblasts (Frangogiannis, 2014). These cells accumulate in granulation tissue, depositing ECM proteins that contribute to scar formation and preserve the structural integrity of the tissue (Zymek et al., 2006). The reparative cells then undergo apoptosis and the wound transitions into the maturation phase and inflammation begins to resolve. The resolution of the inflammatory phase results in the replacement of the vascular granular tissue with a collagen scar (Zymek et al., 2006; Frangogiannis, 2014). As the scar matures, significant collagen cross-linking ensues, increasing the tensile strength of the scar (Czubryt, 2012). This scarring impairs cardiac contractility and relaxation, leading to ventricular remodeling and ultimately heart failure, as well as impairments in electrical signaling synchronicity which can lead to arrhythmogenesis (Miragoli et al., 2007; Czubryt, 2012). It was long thought that, unlike other organs, the heart did not contain endogenous stem cells, and thus, the cells of the heart could not regenerate nor recellularize after damage, rendering the damage and scarring irreversible. In 2003, however, a small, yet biologically important population of endogenous cardiac stem cells were discovered in rats and subsequently in humans (Beltrami et al., 2003; Torella et al., 2007). These findings suggested the heart also has regenerative capacity, albeit limited, and exciting ongoing research, whilst in its infancy, is exploring the impact on cardiac fibrosis.

**Table 1:** Effect of myocardial infarction and idiopathic pulmonary fibrosis on the extracellular matrix in the early and late stages of fibrosis.

**Fibrosis**	**Myocardial Infarction**	**Idiopathic Pulmonary Fibrosis**
Early fibrosis	Migration of monocytes, macrophages and neutrophils to the infarct zone TGF-β stimulated increase in fibroblast chemotaxis Fibroblast proliferation and TGF-β stimulated myofibroblast trans-differentiation Cellular inflammation Cardiomyocyte necrosis Expansion of infarct zone Increased MMP activity; breakdown of collagen network TIMP expression in infarct zone (peaks 48 h post-infarct), allowing collagen activity High Col-III to Col-I ratio Increased systolic and diastolic wall stress, wall thinning and ventricular dilation Deformation of border zone and remote zone tissue Augmented myocardial shortening and increased heart rate leading to hyperkinesis (spasm) of non-infarcted myocardium	Injury to the alveolar epithelium, accompanied by increased epithelial cell apoptosis and senescence Release of inflammatory mediators by epithelial and endothelial cells Extravascular hypercoagulation involving activation of coagulant proteases in association with the formation of a provisional matrix Recruitment of neutrophils, macrophages, lymphocytes and eosinophils to the site of injury Cellular inflammation Activation and migration of fibroblasts to site of injury Increased MMP activity; collagen processing and maturation, localized collagen denaturation High Col-III to Col-I ratio Formation of fibrotic foci comprised of a core of fibroblasts surrounded by hyperplastic or apoptotic/senescent epithelial cells Fibrogenesis emanates from sub-pleural regions of the lower lung
Late fibrosis	Continuous LV dilation Overall LV remodeling and distorted shape Cardiomyocyte hypertrophy (~70% increase in cell volume) Continuing cardiomyocyte necrosis; replacement of myocytes with fibrotic tissue Mural LV wall hypertrophy Decreased Col-III and Col-I ratio Collagen accumulation (mostly type-III and type-I) in infarct zone Scar formation Whole LV hypertrophy	Collagen accumulation (mostly type-III and type-I) in fibrotic lesions Heterogeneous architectural distortion Lower Col-III to Col-I ratio Increased numbers of mast cells, macrophages and lymphocytes Increased ECM stiffness leading to a self-perpetuating fibrosis involving a positive feedback loop Bronchiolisation-activation and migration of basal cells in the conducting airways Honeycombing cystic remodeling Areas of marked fibrosis and scar formation

*Note that the early fibrotic stages of IPF are proposed only, and are still not entirely understood*.

Whilst cardiac fibrosis is largely an adaptive response to myocyte loss and/or cardiac pressure overload, pulmonary fibrosis is a response to epithelial injury. The onset and extent of pulmonary fibrosis depends on the severity and persistence of the initial injury, as well as the regenerative capacity of the epithelium. Inadequate re-epithelialization following injury triggers a cascade of events leading to pathological fibrosis. Increased type II alveolar epithelial cell (AECII) apoptosis and senescence are considered to have important roles in the development of pathological fibrosis in IPF and ARDS (Matute-Bello et al., 1999; Plataki et al., 2005; Hecker et al., 2014; Lopez et al., 2015). Following injury, the stages of fibrosis in cardiac and respiratory disease are similar, as are the cellular and molecular mechanisms that lead to scar formation. However, an important difference distinguishing the fibrosis of IPF to that of many other respiratory and cardiac diseases is the nature and extent of inflammation. Whilst both the innate and adaptive immune systems have roles in IPF, the inflammatory response is far less active than in other forms of fibrosis (Feghali-Bostwick et al., 2007; Gilani et al., 2010; Margaritopoulos et al., 2016). As in cardiac fibrosis, pulmonary fibrosis has consecutive and overlapping fibroproliferative and maturation stages (Table 1). During these stages, epithelial, endothelial, and fibroblast cells undergo migration and/or proliferation in a coordinated process to repair the damaged tissue. The proliferation and transdifferentiation of fibroblasts leads to an increase in the myofibroblast population at the site of injury. These cells facilitate the exaggerated deposition of ECM in aberrant tissue repair, leading to the formation of an irreversible scar. As previously outlined, this scar significantly impairs organ function and ultimately leads to dysfunction and failure. Distinct from cardiac fibrosis, epithelial cells in IPF and ARDS are a major source of mediators essential to the migration, proliferation, and differentiation of resident fibroblasts.

### Inflammation

It is well documented that fibrosis is closely linked to the inflammatory response (Krenning et al., 2010; Biernacka and Frangogiannis, 2011). Emerging evidence suggests that cardiac fibrosis in general is primarily due to complications associated with acute and prolonged inflammation (Biernacka and Frangogiannis, 2011; Van Linthout et al., 2014), suggesting that even low-grade, persistent inflammation is enough to promote cardiac fibrosis. The cellular and molecular events that underpin the inflammatory processes in cardiac disease are complex and poorly understood, with many tissue and disease specific mechanisms. In pulmonary fibrosis however, the role of inflammation is less clear. Certainly, an active inflammatory response following injury to the alveolar epithelium contributes to fibrosis in ARDS. However, the role of inflammation in the development of IPF pathology is less certain. Whether an initial injury event(s) and a subsequent acute inflammatory response occurs is difficult to establish because of the lengthy delay before the diagnosis of IPF is made (Wuyts et al., 2014). The role of inflammation in the progression of fibrosis in IPF has been challenged because anti-inflammatory and immunosuppressive agents are ineffective as therapies (Davies et al., 2003; Richeldi et al., 2003). The current prevailing hypothesis is that IPF is a result of persistent micro-injuries to the alveolar epithelium, rather than a phenomenon driven by inflammation (Selman et al., 2004). Despite this conception, there are increased numbers of inflammatory cells, including mast cells, macrophages, and lymphocytes in the fibrotic lung and circulation of IPF patients (Nagai et al., 2007; Peteranderl et al., 2016). Furthermore, the lung immune cell profiles in IPF and ARDS are similar (Schupp et al., 2015).

Following cardiac tissue injury, inflammatory and immune cells such as monocytes, granulocytes, macrophages, mast cells, dendritic cells, as well as B and T lymphocytes are recruited to the site of injury. These infiltrating cells are responsible for producing and secreting an array of both inflammatory and anti-inflammatory cytokines such as interleukins (e.g., IL-1 and IL-13), tumor necrosis factor-α (TNF-α) and transforming growth factor-β (TGF-β; Afanasyeva et al., 2004; Blyszczuk et al., 2008; Kania et al., 2009; Van Linthout et al., 2014). More recently, these inflammatory cells have been identified to release fibrogenic cytokines and growth factors which promote the fibrotic tissue formation following the initial inflammatory cell infiltrate (Frangogiannis, 2006; Wynn, 2008; Biernacka and Frangogiannis, 2011). Studies aimed at elucidating the role of inflammatory cells in the development of cardiac fibrosis, have focused on the involvement of T helper (Th) lymphocytes, specifically Th1, Th2, and Th17 lymphocytes (Van Linthout et al., 2014). Such studies identify that in cardiac inflammatory wound healing, Th2 lymphocytes act directly and indirectly (via expression of factors such as IL-4, -5, and -13) with fibroblasts to promote myofibroblast activation and suppress matrix metalloproteinase (MMP) activity leading to the accumulation of ECM proteins and formation of fibrotic tissue (Yu et al., 2006; Marra et al., 2009; Wei, 2011; Bailey et al., 2012). Similarly, studies suggest that Th1 lymphocytes have the ability to play both a pro- and anti-fibrotic role. The anti-fibrotic role of Th1 lymphocytes has long been recognized to act via induction of interferon-γ (IFN-γ), which reduces TGF-β expression (Ulloa et al., 1999; Eickelberg et al., 2001), and inhibits IL-4 and -13 (Fairweather et al., 2004), suppressing the formation of fibrotic tissue (Van Linthout et al., 2014). The pro-fibrotic activity of Th1 lymphocytes is also achieved via secretion of IFN-γ, which results in production of pro-fibrotic and pro-inflammatory mediators such as TNF-α (Nathan et al., 1984; Piguet et al., 1989; Chen et al., 2001; Marko et al., 2012). Further studies suggest Th1 cells control activation, migration and differentiation of macrophages, and macrophage dependent monocyte chemoattractant protein-1 expression, leading to further inflammation and fibrosis (Wynn, 2004; Han et al., 2012; Van Linthout et al., 2014). Finally, Th17 cells play an essential role in the development of cardiac fibrosis via the expression of IL-17 (Feng et al., 2009). The pro-fibrotic characteristics of IL-17 are derived from its ability to induce the expression of pro-inflammatory factors, which in turn, promote the recruitment and activation of neutrophils (Ouyang et al., 2008; Pelletier et al., 2010), promote MMP-1 expression in cardiac fibroblasts (Cortez et al., 2007), and induce cardiac fibrosis directly via protein kinase C activation (Liu et al., 2012).

Interactions between inflammatory cells and resident structural cells are likely to contribute to pulmonary fibrosis. Increased numbers of mast cells in the lung of IPF and ARDS patients elicit fibrogenic actions in a manner involving direct contact with fibroblasts (Liebler et al., 1998; Wygrecka et al., 2013). Alternatively activated (M2) macrophages, the predominant macrophage phenotype in the lungs of IPF patients, have an impaired innate immune function but produce fibrogenic mediators such as TGF-β, IL-13, and chemokine (C-C motif) ligand 18 (CCL18; Pechkovsky et al., 2010). The latter mediates a fibrogenic axis between M2 macrophages and fibroblasts in IPF by inducing fibroblast collagen production, which in turn up-regulates M2 macrophage CCL18 expression (Prasse et al., 2006; Stahl et al., 2013). Increased numbers of non-proliferating, antigen-activated T and B cells are also detected in lung tissue and the circulation of IPF patients (Daniil et al., 2005; Marchal-Sommé et al., 2006; Kahloon et al., 2013; Xue et al., 2013). CD8+ T lymphocyte numbers in particular correlate with measures of respiratory disease severity in IPF, implicating a role in pulmonary fibrosis (Daniil et al., 2005). Autoantibodies produced by activated B cells may also contribute to the development of IPF (Donahoe et al., 2015). Whilst most autoantibodies are benign, some, such as those against heat shock protein 70 are pathological (Kahloon et al., 2013). Therapies that reduce autoantibody production, including the B cell targeting rituximab, show potential as treatments for IPF (Donahoe et al., 2015). In lungs of IPF patients, mature lymphocytes, and dendritic cells form aggregates resembling lymphoid follicles, which act autonomously to recruit maturing dendritic cells and recently activated T cells (Marchal-Sommé et al., 2006). One possible explanation for why IPF is refractory to anti-inflammatory therapeutics is that these agents primarily target immature naïve immune cells, not the mature lymphocyte and dendritic cells detected in IPF.

### Pro-fibrogenic mediators

#### Heart

TGF-β has been extensively studied as a mediator of cardiac fibrosis. The TGF-β signaling cascade is an essential component of the inflammatory and reparative phases. TGF-β has pleiotropic effects on cell types important to cardiac injury, repair and remodeling (Dobaczewski et al., 2011). Latent TGF-β stores exist in the myocardium for rapid activation following cardiac insult (Frangogiannis, 2014; Kong et al., 2014). The differentiation of fibroblasts into myofibroblasts involves the release of TGF-β, which induces α-smooth muscle actin expression, as well as the secretion and deposition of ECM proteins and the removal of the pro-inflammatory mediators which inhibit myofibroblast conversion. The production of reactive oxygen species, induction of matricellular proteins and activation of proteases, contribute to activation of preformed TGF-β at the site of infarction. Furthermore, platelets, leukocytes and fibroblasts migrate to the site of cardiac injury and contribute to the increase in TGF-β levels at the site of injury immediately after infarction. However, the abundance of circulating pro-inflammatory mediators at the early stage post-infarction is likely to reduce cellular responsiveness to TGF-β delaying cellular differentiation to myofibroblasts and the secretion and deposition of ECM proteins until the infarct is clear of necrotic cells and matrix debris conversion (Dobaczewski et al., 2011; Westermann et al., 2011; Frangogiannis, 2014). Upon completion of the inflammatory process which clears the infarct, TGF-β signaling is then able to promote myofibroblast differentiation, thus activating molecular signaling that releases ECM proteins such as collagen, and promotes matrix preservation as well as upregulating the synthesis of protease inhibitors (Frangogiannis, 2014).

Platelet-derived growth factor (PDGF) has a well-established role in regulating fibrosis and angiogenesis via interactions with PDGF receptor alpha (PDGFR-α) and beta (PDGFR-β). Activation of these two receptors induces fibroblast migration, proliferation and activation (Yu et al., 2001; Raines, 2004). Both PDGF receptors have been shown to have regulatory roles in scar formation. For example, over-expression of the PDGFR-α ligand, PDGF-C, has been shown to induce increased cardiac fibrosis, collagen deposition, vascular defects, and myocardial hypertrophy (Ponten et al., 2003). Moreover, overexpression of the PDGFR-β ligand, PDGF-D, results in cardiac fibrosis followed by dilated cardiomyopathy and heart failure (Ponten et al., 2003). PDGFR-β has also been shown to have an additional role in regulating vascular maturation, by acquiring a mural muscle coat for the infarct's micro-vessels (Gerhardt and Betsholtz, 2003; Ponten et al., 2003; Zymek et al., 2006). Furthermore, genetic disruption of PDGFR-β has been shown to result in increased microvascular leaks, lethal hemorrhages and edema in late embryogenesis (Leveen et al., 1994; Zymek et al., 2006). Inhibition of PDGFR-β activity resulted in impaired vascular maturation in healing infarcts whereas PDGFR-α inhibition had no effect on vessel maturation, but significantly reduced the amount of collagen deposition at the site of infarct (Ponten et al., 2003; Zymek et al., 2006).

**Figure 1:**
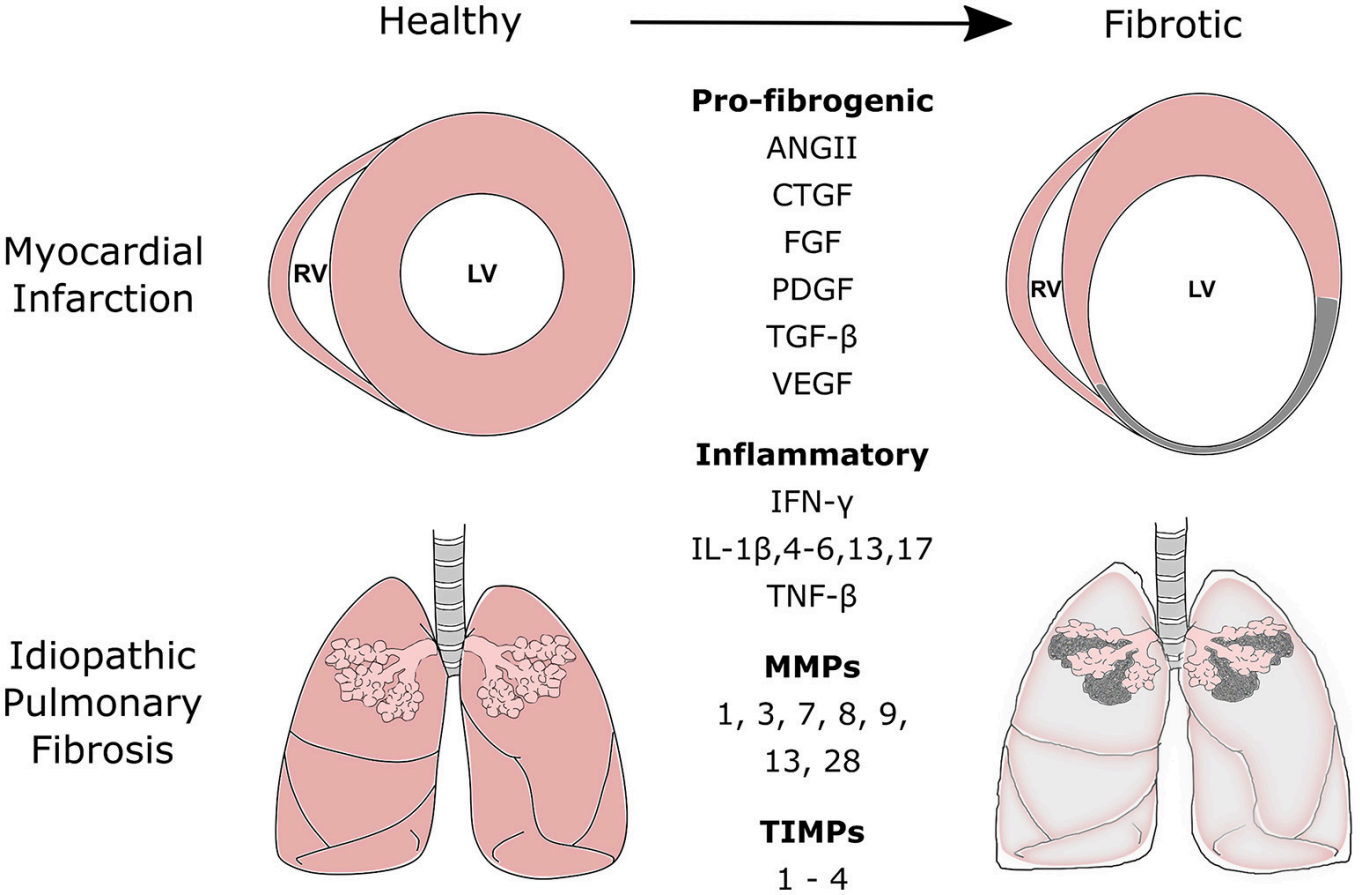
Commonly secreted pro-fibrogenic growth factors, inflammatory proteins, matrix metalloproteinases (MMPs), and tissue inhibitors of metalloproteinases (TIMPs) during the fibrotic processes of myocardial infarction and idiopathic pulmonary fibrosis. Fibrotic disease of the heart and lung is the result of a range of cellular and molecular responses activated by tissue injury. The fibrotic process is tightly regulated and involves three distinct phases: the inflammatory, proliferative, and maturation phase. During the inflammatory and proliferative phases, a number of pro-fibrogenic, and inflammatory mediators are released to recruit and activate reparative mesenchymal cells such as fibroblasts and myofibroblasts. These cells aid scar formation and maintains the structural integrity of the tissue. MMPs and TIMPs are released by fibroblasts. Their release can be further mediated by various chemokines, cytokines, and growth factors released during the remodeling process. MMPs and TIMPs work in concert to control the remodeling and degradation of extracellular matrix proteins at the site of injury. ANGII, Angiotensin II; CTGF, Connective Tissue Growth Factor; FGFs, Fibroblast Growth Factors; PDGF, Platelet-Derived Growth Factor; TGF, Transforming Growth Factor; VEGF, Vascular Endothelial Growth Factor; IFN-γ, Interferon-γ; IL, Interleukin; TNF, Tumor Necrosis Factor.

The renin-angiotensin system is another well-established regulator of cardiac fibrosis (Leask and Abraham, 2004; Haudek et al., 2010; Frangogiannis, 2014; Kong et al., 2014). Angiotensin II (AngII) is the primary effector molecule of renin-angiotensin system and is formed by enzymatic cleavage of angiotensinogen into angiotensin I by renin, which is subsequently cleaved into AngII by angiotensin converting enzyme (ACE; Leask and Abraham, 2004; Haudek et al., 2010; Kong et al., 2014). Myofibroblasts and inflammatory cells such as macrophages are known sources of renin and ACE, and thus are capable of modulating circulating levels of AngII (Katwa et al., 1997; Haudek et al., 2010). *In vitro* studies have demonstrated that AngII can increase fibroblast activation and proliferation, collagen production and cardiomyocyte hypertrophy and apoptosis (Sadoshima and Izumo, 1993; Mehta and Griendling, 2007; Xu et al., 2013; Frangogiannis, 2014). Furthermore, *in vivo* rodent studies have demonstrated that the intravenous administration of AngII results in significant myocardial fibrosis, and that serum and tissue levels of AngII were elevated in animals with pressure-overloaded hearts (Misaka et al., 2013). Moreover, AngII inhibition using either ACE inhibitors or AngII type I receptor blockers significantly improved cardiac function in patients with hypertension as well as animal models of myocardial infarction remodeling (Patterson, 2003; Liu et al., 2015). Additionally, the use of these AngII-inhibiting agents induced the regression of cardiac remodeling (Figure 1).

#### Lung

TGF-β is also a central mediator of pulmonary fibrosis. The expression of TGF-β and/or its receptors are increased in lung epithelial cells, macrophages and fibroblasts in IPF and non-resolving ARDS (Khalil et al., 2001; Fahy et al., 2003). Increased TGF-β affects the alveolar-capillary barrier by disturbing fluid dynamics and inducing AECII apoptosis. Conversely, TGF-β inhibits fibroblast apoptosis, a key step in the resolution of wound repair responses. Furthermore, TGF-β also expands the mesenchymal compartment by inducing epithelial-mesenchymal transdifferentiation and fibroblast proliferation, cytokine production and their differentiation into myofibroblasts. The genes encoding myofibroblast-associated contractile proteins, such as α-smooth muscle actin and the ECM molecules, pro-collagens-I and -III, all contain TGF-β response elements in their promoters and are regulated by Smad signaling pathways. In support of its role in pulmonary fibrosis, the selective gene transfer of TGF-β into AECs induces fibrotic responses *ex vivo*, whereas blocking TGF-β-signaling in experimental models of lung injury attenuates pulmonary edema and fibrosis (Nakao et al., 1999; Lee et al., 2004; Peters et al., 2014; Dadrich et al., 2016). In an acute manner, the addition of ectopic TGF-β to alveolar airspaces *in vivo* or lung slices *ex vivo* augments fluid retention by impairing Na^2+^ and fluid transport (Peters et al., 2014).

TGF-β is a validated drug target in models of pulmonary fibrosis. However, the direct targeting of TGF-β or its canonical Smad signaling pathways is unfeasible as an anti-fibrotic therapy because of the essential role of TGF-β in immunity and tissue homeostasis. However, down-stream mediators of TGF-β, or modifiers of TGF-β effector function, are plausible alternative therapeutic targets. TGF-β up-regulates the expression of fibroblast growth factors (FGFs), a family of soluble protein mediators involved in lung development and tissue homeostasis. Members of the FGF family, including FGF-1, FGF-2, and FGF-9, along with their receptors, are increased in fibrotic lung and implicated in pulmonary fibrosis (Coffey et al., 2013; MacKenzie et al., 2015). The proliferative effects of TGF-β on pulmonary fibroblasts are mediated by autocrine actions of FGF (Khalil et al., 2005). Interestingly, FGF when applied ectopically, can be protective in experimental pulmonary fibrosis (Fang et al., 2010). These opposing roles are possibly explained by its dual roles in epithelial reparation and fibroblast migration/proliferation. The PDGF family of TGF-β-regulated cytokines are also potent mitogens, contributing to the expansion of fibroblasts in the fibro-proliferative phases of IPF and ARDS (Kelly et al., 2003; Piednoir et al., 2012; Wollin et al., 2015). The expression of PDGF and its receptors are increased in the epithelium, macrophages and fibroblasts of fibrotic lung tissue, and targeting PDGF signaling by either pharmacological or genetic approaches in experimental fibrosis is protective (Homma et al., 1995; Yoshida et al., 1999; Dadrich et al., 2016). The efficacy of the FDA-approved treatment for IPF, nintedanib, is due to its inhibition of PDGF, FGF and vascular endothelial growth factor-signaling pathways (Fala, 2015). Connective tissue growth factor is also a downstream mediator of TGF-β, which has been shown to mediate fibrosis in several organs, including the heart and lung (Lipson et al., 2012).

Interactions between the signaling pathways of TGF-β and AngII (Uhal et al., 2007), amphiregulin (Lee et al., 2014), wingless/int, or IL-13 (Murray et al., 2008) all contribute to pulmonary fibrosis. The cross-talk between the TGF-β and AngII pathways is of particular importance, as they act synergistically in the development and progression of pulmonary fibrosis. The role of AngII in pulmonary fibrosis is evidenced by studies that show ACE inhibitors or AT1R-selective antagonists attenuate experimental pulmonary fibrosis (Uhal et al., 2012). Furthermore, polymorphisms in the gene encoding ACE are associated with IPF and ARDS susceptibility or outcome (Marshall et al., 2002; Uh et al., 2013). Fibroblasts and AECs in lung tissue of IPF patients synthesize AngII *de novo*, by expressing components of the renin-angiotensin system, including the AngII precursor, angiotensinogen (Li et al., 2006). AngII contributes to pulmonary fibrosis by stimulating pulmonary fibroblast proliferation via angiotensin receptor 1 (ATR1), involving autocrine TGF-β signaling (Marshall et al., 2000). AngII also induces apoptosis in AECIIs via ATR1 (Papp et al., 2002). The interactions between the AngII and TGF-β pathways occurs in a reciprocal manner. In pulmonary fibroblasts, TGF-β is a potent stimulus of angiotensinogen and ATR1 expression, whereas AngII up-regulates TGF-β expression (Abdul-Hafez et al., 2009). The synergy between both pathways suggests that the targeting of AngII synthesis or function is a potential treatment for IPF (Figure 1).

### Regulation of the extracellular matrix

In fibrosis, the ECM is substantially thickened by an increase in the abundance of fibrillar collagens I and III. Fibril formation is facilitated by increases in fibronectin, a nucleator of fibrillogenesis (i.e., collagen fibril assembly), whereas collagen XII and XIV have roles in collagen distribution and organization (Fukuda et al., 1998). Crosslinking of collagen catalyzed by lysyl oxidase and glycation contributes to the increased rigidity and stiffness of fibrotic tissue (Tzortzaki et al., 2006; Barry-Hamilton et al., 2010). Interestingly, alterations in lysyl oxidase family members have been linked to IPF (Barry-Hamilton et al., 2010; Aumiller et al., 2017). Collectively, these biophysical changes to the ECM in a disease context possibly perpetuate fibrosis as the proliferative and contractile responses of pulmonary fibroblasts increase when they are attached to rigid matrices (Marinkovic et al., 2013; Zhou et al., 2013). Such a detrimental positive feedback loop involving increased ECM stiffness is thought to contribute the distinctive expansive pulmonary fibrosis of IPF (Marinkovic et al., 2013; Zhou et al., 2013).

Homeostatic balance of the ECM network involves complex modulation by multiple factors, and includes the ongoing process of production and deposition of ECM proteins. Two of the major ECM modulators are localized non-structural proteins that facilitate the rearrangement of the ECM under physiological and pathological conditions. These proteins are the MMPs which degrade ECM proteins and their inhibitors: tissue inhibitors of metalloproteinases (TIMPs). MMPs and TIMPs are released throughout the process of tissue healing by fibroblasts, and they work together to control the remodeling and degradation of the existing ECM in and around the site of injury (Fan et al., 2012; Rienks et al., 2014). Several chemokines, cytokines and growth factors have been shown capable of regulating the production of MMPs and TIMPs by fibroblasts. For example, pro-inflammatory cytokines such as TNFα and IL-1β can induce the transcription of several MMPs along with TIMP-1 and -2 in the myocardium (Fan et al., 2012).

MMPs are synthesized and secreted as inactive zymogens (pro-MMPs) which are activated by removal of an amino-terminal propeptide domain, which exposes a catalytic domain (Vanhoutte and Heymans, 2010; Fan et al., 2012). There are ~26 identified MMPs to date, and of these MMP-1, -2, -3, -8, -9, -12, -13, and -28 have been implicated in myocardial remodeling (Fan et al., 2012). MMP-1, -8, and -13 cleave native (fibrillar) forms of collagen types I, II and III, whereas the gelatinolytic MMP-2 and -9 cleave collagen types I, IV, and V (Fan et al., 2012). MMPs are additionally responsible for the release of growth factors from the ECM, cleavage of receptors for these growth factors at the cell's surface and activation of other MMPs (Vanhoutte and Heymans, 2010). In addition, the membrane-type MMP (MT1-MMP/MMP14), which cleaves several ECM proteins, has been highlighted to be of importance in the heart, as a reduction in MT1-MMP improves survival and heart function post myocardial infarction in mice, whilst overexpression lowers survival and function (Zavadzkas et al., 2011). In IPF and ARDS, the levels of MMP-1, -3, -7, -8, -9, -13, and/or -28 in lung tissue are increased, correlating with disease severity (Selman et al., 2000; Henry et al., 2002; Fligiel et al., 2006; Craig et al., 2015). Furthermore, several membrane- type MMPs have been shown to be associated with IPF. MT1-MMP and MT2-MMP are increased in the lungs of IPF patients and murine lung fibrosis models, and MT3-MMP was expressed by fibroblasts and AECs in IPF (Craig et al., 2015; Pardo et al., 2016). MT6-MMP was also shown to be decreased in lung tissue of patients with IPF (Pardo et al., 2016). Other proteases involved in collagen remodeling including urokinase plasminogen activator and fibroblast activation protein α are increased in the lung interstitium of IPF patients (Acharya et al., 2006; Schuliga et al., 2017). A potential mechanism by which MMPs and other matrix degrading proteases contribute to pulmonary fibrosis is by remodeling collagen into forms that evoke fibrogenic actions. MMP-1, -8, and -13 and fibroblast activation protein α are collagenolytic enzymes that cleave and subsequently release native collagen I and III fibrils. The resulting collagen fragments retain the triple helical conformation that binds α2β1 integrin on pulmonary fibroblasts to augment proliferation (Schuliga et al., 2009). Conversely, native collagen I and III bind α2β1 integrin in a different manner that suppresses pulmonary fibroblast proliferation (Schuliga et al., 2009, 2010). The pericellular proteolytic denaturation of collagen immediately surrounding pulmonary fibroblasts possibly preserves the proliferative, synthetic phenotype of fibroblasts with little impact on net tissue stiffness. The spread of fibrosis from discrete regions of the lower lung, as happens in IPF, may occur because of pericellular proteolysis at the leading edge, where fibroblast proliferation is most active (Acharya et al., 2006; Nkyimbeng et al., 2013). Asides from their direct role in ECM remodeling, MMPs possibly contribute to fibrosis by inducing epithelial-mesenchymal transdifferentiation, fibrocyte infiltration, and the polarization of alveolar macrophages into a M2 phenotype via activation of fibrogenic cytokines (Craig et al., 2015).

TIMPs, as their name suggests, inhibit MMP activity by binding to active sites on their respective MMPs, thus preventing them from cleaving ECM components (Vanhoutte and Heymans, 2010). TIMP-2, -3, and -4 are expressed in the healthy heart, whilst TIMP-1 expression is low, although it is upregulated under pathological conditions (Fan et al., 2012). It is becoming increasingly apparent however that TIMPs have MMP-independent functions including the regulation of apoptosis, cell survival, growth, migration, differentiation, angiogenesis, and inflammation (Vanhoutte and Heymans, 2010). Adenovirus-mediated overexpression of TIMP-1, -2, -3, or -4 in cardiac fibroblasts increases proliferation and myofibroblast differentiation, whereas TIMP-2 overexpression alone increases collagen production, and TIMP-3 induces apoptosis (Lovelock et al., 2005). TIMPs are also important regulators of ECM turnover in the lung. However, information regarding TIMP levels and distribution in fibrotic respiratory disease is either limited or inconsistent. To our knowledge, no study has assessed TIMP levels in human fibrotic ARDS, besides measurements in bronchoalveolar lavage fluid (BALF), tracheal aspirates or serum, none of which accurately reflect tissue levels (Miller et al., 2006). Initial studies indicated that ECM accumulation in IPF is a consequence of increased TIMP production and a resulting non-degrading collagen environment. Immunohistochemical analysis of lung tissue of IPF patients showed an association between TIMP-2 antigen and fibroblasts, whereas TIMP-1 antigen was present in interstitial macrophages and TIMP-3 and -4 antigens were confined to the epithelium (Selman et al., 2000; Garcia-Alvarez et al., 2006). Garcia-Alvarez et al. also detected TIMP-3 in fibrotic regions (including fibroblasts) of IPF lung tissue *in situ*, and showed that IPF-derived pulmonary fibroblasts expressed higher levels of TIMP-1, -2, and -3 *in vitro* characterized by compared to fibroblasts characterized by from control donors (Garcia-Alvarez et al., 2006). However, Nkyimbeng et al. (2013) recently showed that mRNA and protein levels of TIMPs in the lung of IPF patients and controls were similar, whereas the levels of MMP-1, -7, and -13 were higher in IPF. They also showed that MMP-1 and -13 antigenicity in lung of IPF patients' *in situ* overlap with collagenolytic activity in certain regions of fibrosis, particularly the airways where honeycombing cysts develop. Based on these findings, the authors suggested that the levels of TIMPs in regions of IPF lung are inadequate to counter increased collagenolytic MMP production. However, despite these local increases in collagenolytic activity, overwhelming matrix production in IPF results in a net increase in ECM. Another potentially important role of TIMPs in IPF and ARDS is the regulation of inflammatory responses that drive fibrosis. TIMP-3 in particular has important roles in M2 macrophage polarization in lung injury and disease (Gill et al., 2010, 2013).

## Current therapies for fibrosis

Current treatments for heart failure include administration of ACE inhibitors, β-blockers and spironolactone, which taken together, target vasodilatation, heart rate and diuresis. This standard clinical combination of therapeutics has been shown to reduce death following heart failure, but does not cure or reverse it. As it stands, there are currently no viable therapeutic options for heart failure (Breckenridge, 2010). Traditionally, cardiac fibrosis has not been a major therapeutic target in heart disease, however it is now believed that targeted cardiac fibrosis therapy may reduce the progression of heart failure. The potential therapeutic benefits of inhibition of the TGF-β pathway via targeting of Smad transcription factors are under investigation at varying stages of the scientific process (Roubille et al., 2014). For example targeting Smad3 has been shown to exert antifibrotic effects, and does so by attenuating cardiac remodeling (Bujak et al., 2007). Moreover, other pathways linked to the TGF-β signaling pathways have been identified as potential therapeutic targets in a number of pulmonary fibrotic conditions (Roubille et al., 2014). These investigations are currently in the early pre-clinical stage, and thus, therapies for fibrosis attenuation in heart failure are not yet available.

Until 2014, there was no effective IPF treatment. Glucocorticoids were used as standard therapy for IPF, but showed no appreciable effect on clinical outcome (Selman et al., 2004). Whilst the anti-oxidant, *N*-acetylcysteine combined with glucocorticoids decelerates loss of lung function for IPF patients, there is no effect on mortality rates (Demedts et al., 2005). However, two relatively recent FDA-approved therapies, pirfenidone, and nintedanib, reduce the rate of decline in lung function and increase survival, in some patients, albeit modestly (King et al., 2014; Richeldi et al., 2014). The mechanism underlying the anti-fibrogenic and inflammatory actions of pirfenidone are not well understood, but are thought to involve suppression of TGF-β and TNF-α at the translational level (Nakazato et al., 2002). Nintedanib, a receptor tyrosine kinase inhibitor, inhibits the actions of FGF, PDGF and vascular endothelial growth factor (Fala, 2015) with IC_50_ values <100 nM. The polypharmacology of nintedanib, targeting several down-stream signaling cascades involved in angiogenesis and fibroblast proliferation, differentiation and collagen production, is likely to contribute to its effectiveness as an IPF therapy. Whilst the effects of both pirfenidone and nintedanib are small and with significant side effects, they do provide clinicians and patients hope that IPF is treatable. Given the relative success of pirfenidone in IPF, recent trials have investigated its use in animal models of cardiac fibrosis. These studies demonstrated a reduction in ventricular remodeling, and reduced cardiac fibrosis in a canine and murine model of heart failure, respectively (Lee et al., 2006; Wang et al., 2013; Yamagami et al., 2015). Clinical trials for Pirfenidone, however, have yet to be undertaken in human cardiac disease. Furthermore, a recent clinical trial investigating a monoclonal antibody, FG-3019, which interferes with the action of connective tissue growth factor was shown to be safe and well-tolerated in IPF patients, and demonstrated good outcomes in pulmonary function and extent of fibrosis (Raghu et al., 2016). Larger phase two clinical trials are currently underway (NCT01890265).

The mainstay treatment for ARDS has also been glucocorticoids. However, whilst glucocorticoids were once thought to reduce fibroproliferation in ARDS, recent studies show that they have no effect on mortality rates for patients who display ongoing ARDS criteria 7 days after injury, although glucocorticoids improve lung function and shorten time on mechanical ventilation (Meduri et al., 1994; Hudson and Hough, 2006). A major negative effect of glucocorticoids treatment for persistent ARDS is the development of intensive care unit-acquired weakness (Zorowitz, 2016). However, the beneficial effects of glucocorticoids on overall clinical improvement appear to outweigh their harmful neuromuscular effects which include muscle wasting (Meduri et al., 2016). In the United States, ARDS mortality, which is closely related to the degree of fibrosis, was reduced from ~40 to ~25% in 5 years from 2000 (National Heart, Lung, and Blood Institute Acute Respiratory Distress Syndrome (ARDS) Clinical Trials Network et al., 2006). This reduction was mainly a result of mechanical ventilation therapy and improved management practices, rather than the development of new drug-interventions. Whilst numerous pharmacological treatments have been evaluated for ARDS in clinical trials, none have shown any real measurable therapeutic effect, except in small subgroups of patients with specific causes of lung injury.

An exciting advance in the treatment of chronic fibrosis may be the use of stem and progenitor cell-based therapies in which substantial pre-clinical, and early phase clinical studies suggest efficacy in multiple organ systems. Pre-clinical trials of cell-based therapies in animal models of heart failure and IPF have demonstrated substantial reductions in fibrotic tissue in the left ventricle and lungs, respectively, and have led to several Phase I/II clinical trials (Toonkel et al., 2013; Le et al., 2017). These studies include the use of bone marrow, placenta, and adipose tissue derived mesenchymal stem cells, with one study investigating autologous lung stem cells (NCT02745184). A 2017 systematic review of autologous stem cell therapy in ischemic heart disease and heart failure demonstrated that of the 21 randomized controlled trials included, mortality was significantly reduced at 12 months post-treatment (4.8 vs. 15.4% in non-treated) with no major adverse events (Fisher et al., 2017). Although, fibrosis or therapeutic mechanisms were not investigated in this review, preclinical studies suggest that reduction in left ventricular remodeling could be playing a role. Authors cautioned, however, that the included studies were small and despite the promising results, larger clinical studies are needed. The majority of the pulmonary fibrosis cell-based therapy studies are still ongoing, however two phase-1 trials have recently been published and suggest allogeneic placental and bone marrow derived mesenchymal stem cells were feasible and well tolerated in IPF patients (Chambers et al., 2014; Glassberg et al., 2017) and future studies to determine efficacy are warranted.

## Summary

Cardiac and pulmonary fibrosis differ markedly in etiology and outcome of disease. Such divergences relate to the specific structure and function of the heart and lungs. However, many similarities between cardiac and pulmonary fibrosis exist, including shared biochemical pathways and cellular processes involving inflammatory cells, fibroblasts and ECM. A mutual pathological theme for both forms of fibrosis involves the ECM. In a detrimental feed-forward loop, increased accumulation of stiffened ECM appears to perpetuate an ongoing fibrogenic response. In cardiac fibrosis, the adaptive accumulation and remodeling of fibroblast-derived ECM contributes to pressure overload, subsequent cardiomyocyte death and the formation of more ECM as replacement. With the exception of myocyte participation, a comparable self-perpetuating cycle occurs in the pulmonary fibrosis of IPF, involving the progressive accumulation of fibroblasts and ECM. Although, further investigations are required to fully understand the mechanisms of chronic fibrosis, given the similarities between cardiac and pulmonary fibrosis, investigating targets and testing future treatments in both organs seems justifiable and may lead to better treatment opportunities for diseases such as CVD and IPF.

## Author contributions

LM, MS, NM, DK, and AB were involved in the conception and design of the review article. LM, MS, and NM drafted the manuscript and figures. SH, JB, and DW assisted with drafting the manuscript. All authors reviewed and edited the final manuscript, gave final approval of the version to be published, and agree to be accountable for all aspects of the work.

### Conflict of interest statement

The authors declare that the research was conducted in the absence of any commercial or financial relationships that could be construed as a potential conflict of interest.

## Abbreviations

ACE: Angiotensin Converting Enzyme
AEC: Alveolar Epithelial Cell
AngII: Angiotensin II
ARDS: Acute Respiratory Distress Syndrome
ATR1: Angiotensin Receptor 1
BALF: Bronchoalveolar Lavage Fluid
CVD: Cardiovascular Disease
ECM: Extracellular Matrix
FGFs: Fibroblast Growth Factors
IFN-γ: Interferon-γ
IL: Interleukin
IPF: Idiopathic Pulmonary Fibrosis
MMPs: Matrix Metalloproteinases
PDGF: Platelet-Derived Growth Factor
PDGFR: PDGF Receptor
TGF-β: Transforming Growth Factor-β
Th: T helper
TIMPs: Tissue Inhibitors of Metalloproteinases
TNF-α: Tumor Necrosis Factor-α
UIP: Usual Interstitial Pneumonia.
